# Immunological characterization of a *gidA* mutant strain of *Salmonella* for potential use in a live-attenuated vaccine

**DOI:** 10.1186/1471-2180-12-286

**Published:** 2012-11-30

**Authors:** Daniel C Shippy, Amin A Fadl

**Affiliations:** 1 Department of Animal Sciences, University of Wisconsin-Madison, 1675 Observatory Dr, Madison, WI, 53706, USA

**Keywords:** Salmonella, GidA, Vaccine, Immune responses, Mouse model

## Abstract

**Background:**

*Salmonella* is often associated with gastrointestinal disease outbreaks in humans throughout the world due to the consumption of contaminated food. Our previous studies have shown that deletion of glucose-inhibited division gene (*gidA*) significantly attenuated *Salmonella enterica* serovar Typhimurium (STM) virulence in both *in vitro* and *in vivo* models of infection. Most importantly, immunization with the *gidA* mutant protected mice from a lethal dose challenge of wild-type STM*.* In this study, we further characterize the *gidA* mutant STM strain for potential use in a live-attenuated vaccine.

**Results:**

The protective efficacy of immunization with the *gidA* mutant was evaluated by challenging immunized mice with a lethal dose of wild-type STM*.* Sera levels of IgG2a and IgG1, passive transfer of sera and cells, and cytokine profiling were performed to study the induction of humoral and cellular immune responses induced by immunization with the *gidA* mutant strain. Additionally, a lymphocyte proliferation assay was performed to gauge the splenocyte survival in response to treatment with STM cell lysate. Mice immunized with the *gidA* mutant strain were fully protected from a lethal dose challenge of wild-type STM. Naïve mice receiving either cells or sera from immunized mice were partially protected from a lethal dose challenge of wild-type STM*.* The lymphocyte proliferation assay displayed a significant response of splenocytes from immunized mice when compared to splenocytes from non-immunized control mice. Furthermore, the immunized mice displayed significantly higher levels of IgG1 and IgG2a with a marked increase in IgG1. Additionally, immunization with the *gidA* mutant strain evoked higher levels of IL-2, IFN-γ, and IL-10 cytokines in splenocytes induced with STM cell lysate.

**Conclusions:**

Together, the results demonstrate that immunization with the *gidA* mutant strain protects mice by inducing humoral and cellular immune responses with the humoral immune response potentially being the main mechanism of protection.

## Background

*Salmonella* is an enteric pathogen causing major public health problems throughout the world due to the consumption of contaminated food. Nontyphoidal *Salmonella* species, like *Salmonella enterica* serovar Typhimurium (STM), are the leading cause of hospitalization and death among the major foodborne pathogens [1]. Antibiotic resistance by *Salmonella* is dramatically increasing, so the development of an effective vaccine remains a global health priority [2,3].

Creating a safe and immunogenic vaccine strain is the biggest challenge in developing an effective live-attenuated *Salmonella* vaccine [4]. Several *Salmonella* vaccines, including whole-cell killed and live vaccines, have been developed with variable success [5,6]. These vaccines either required repeated administration or induced insufficient immune responses for long-lasting protection against lethal challenges with virulence *Salmonella* strains [7]. Many *Salmonella* vaccine strains carry deletion mutations affecting metabolic functions or virulence factors [8]. Several mutant strains of *Salmonella* have been investigated in the pursuit to develop optimal immune responses [9-11]. Our approach in constructing a live-attenuated *Salmonella* vaccine strain is to create a mutant defective in tRNA modification [12]. This strategy enables our vaccine strain to express multiple virulence factors at a significantly reduced level in order to obtain a safe and immunogenic vaccine candidate.

Glucose-inhibited division (GidA) protein (also known as MnmG) was first described in *Escherichia coli,* where deletion of *gidA* resulted in a filamentous morphology when grown in a rich medium supplemented with glucose [13]. Further studies showed GidA is a flavin dinucleotide (FAD) binding enzyme involved in the fruiting body development of *Myxococcus xanthus*[14]. Furthermore, GidA has been shown to be a tRNA modification methylase in *E. coli* that forms a heterodimeric complex with MnmE (also known as TrmE) to catalyze the addition of a carboxymethylaminomethyl (cmnm) group at the 5 position of the wobble uridine (U34) of tRNAs [15-19]. Most importantly, deletion of *gidA* has been shown to attenuate the pathogenesis of some bacteria including *Pseudomonas syringae, Aeromonas hydrophila, Streptococcus pyogenes,* and *Pseudomonas aeruginosa*[20-23].

Our previous studies suggest a role for GidA in the regulation of *Salmonella* virulence and cell division [12,24]. In our initial study, the *gidA* mutant was attenuated *in vitro* and showed a significant decrease in ability to invade T84 intestinal epithelial cells as well as a significant decrease in ability to replicate and produce cytotoxic affects on macrophages. Furthermore, global transcriptional and proteomic profiling indicated a significant down-regulation in numerous genes and proteins involved in *Salmonella* pathogenesis [12]. Most importantly, the *gidA* mutant was attenuated in mice as shown by a significant increase in 50% lethal dose (LD_50_), reduced systemic bacterial survival, defective in the induction of inflammatory cytokines and chemokines, and reduced severity of histopathological lesions in the liver and spleen. Additionally, mice immunized with the *gidA* mutant were protected from a lethal dose challenge of wild-type (WT) STM [12].

In this study, we examined the relative contribution of the humoral and cellular immune responses in the overall protective mechanism afforded by immunization with the *gidA* mutant STM strain to further evaluate it as a candidate for use in a live-attenuated vaccine. The protective efficacy of immunization with the *gidA* mutant was evaluated by challenging immunized mice with a lethal dose of WT STM*.* Sera of control and immunized mice were tested for levels of IgG1 and IgG2a to gauge the Th1 and Th2 responses to *gidA* immunization. Additionally, sera and cell culture supernatant were used to determine the level of induction of Th1 (IL-2 and IFN-γ) and Th2 (IL-4 and IL-10) cytokines in control and immunized mice. Passive transfer studies were performed to evaluate the role of humoral and cell mediated immunity afforded by immunization with the *gidA* mutant vaccine strain. A lymphocyte proliferation assay was used to determine the ability of control and immunized murine splenocytes to respond to treatment with STM cell lysate. Taken together, these data indicate the *gidA* mutant vaccine strain protects mice by inducing humoral and cellular immune responses with the humoral immune response being the primary mechanism of protection.

## Methods

### Bacterial strains and growth conditions

The WT and *gidA* mutant *Salmonella enterica* serovar Typhimurium (STM) 14028 strains are described in [12]. The organisms were grown in Luria-Bertani (LB) broth and on LB agar plates in the presence of nalidixic acid (150 μg/ml) or kanamycin (50 μg/ml). The bacteria were cultivated at 37°C with shaking at 225 rpm. Bacteria were harvested by centrifugation (5,000 rpm for 10 min), washed twice with PBS, and resuspended in a minimal amount of PBS.

### Immunization of mice

Female BALB/c mice, 6–8 weeks old, were obtained from Harlan Laboratories (Indianapolis, IN). All animal procedures were approved by the University of Wisconsin-Madison Animal Care and Use Committee. Mice were kept under specific pathogen-free conditions in filter-topped cages and provided with food and water ad libitum. Mice were inoculated via the intraperitoneal (i.p.) route with either 1 x 10^3^ CFU of the *gidA* mutant STM strain, or sterile PBS. The chosen time points for the assays in this study are 7 and 42 days after immunization. These time points were chosen to gauge the immune response to the *gidA* mutant STM strain at the early stage of infection and at the time of challenge. At these time points, mice were sedated with isoflurane (Abbott Laboratories, North Chicago, IL) and bled for sera which were used to profile the Th1 and Th2 cytokines, determine the IgG subclasses, and used in the passive transfer experiment. The spleens were removed and these cells were used for the cell population analysis, lymphocyte proliferation assay, Th1 and Th2 cytokine profiling, and the passive transfer experiment. At the 42 day time-point, selected mice that had been injected with PBS and the *gidA* mutant STM strain were challenged with a lethal dose (1 x 10^5^ CFU) of WT STM. Morbidity and mortality of these animals were monitored for 30 days after challenge. Mice suffering from lethal salmonellosis as determined by severe hunched posture, labored breathing, apathy, and ruffled fur were euthanized to prevent unnecessary suffering.

### Splenic bacterial counts

The enumeration of bacteria from the spleen was performed as previously described [12]. Briefly, spleen samples of 0.1 g were removed from mice inoculated with sterile PBS or the *gidA* mutant STM strain, homogenized in 1 ml PBS, and serial dilutions of the homogenate were plated on *Salmonella-Shigella* (SS) and LB agar plates. The plates were incubated at 37°C for 24 hours and colonies were counted. Bacteria were enumerated by determining the CFU in duplicate, and expressed as CFU/ml.

### Flow cytometric analysis

Spleens were removed from mice inoculated with sterile PBS or the *gidA* mutant STM strain. The spleens were homogenized in RPMI media supplemented with 2% fetal bovine serum (FBS), filtered through a 70 μm strainer, and the red blood cells were lysed with Pharm Lyse cell lysis buffer (BD Bioscience, Franklin Lakes, NJ). The spleen cells were washed twice with PBS supplemented with 2% FBS, filtered through a 70 μm strainer, and counted on a hemocytometer. Approximately 1 x 10^6^ cells were placed in each tube, and incubated with mouse CD16/CD32 monoclonal antibodies (0.25 μg/100 μl) (BD Bioscience) for 15 min at room temperature to block antibody binding to mouse Fc-γ receptors. The cells were washed twice with PBS supplemented with 2% FBS and incubated with either anti-CD4 antibody conjugated to PE-Cy5 (0.20 μg/100 μl) or anti-CD8 antibody conjugated to PE-Cy7 (0.30 μg/100 μl) and anti-CD44 antibody conjugated to fluorescein isothiocyanate (FITC) (0.20 μg/100 μl) and anti-CD62L antibody conjugated to phycoerythrin (PE) (0.10 μg/100 μl). After incubation, the cells were washed once with PBS supplemented with 2% FBS and fixed with 1% formaldehyde. Analysis was performed at the University of Wisconsin-Madison Carbone Cancer Center Flow Cytometry Laboratory using a LSRII flow cytometer and FlowJo software (Tree Star Inc., Ashland, OR).

### ELISA

Initially, a whole-cell *Salmonella* enzyme-linked immunosorbent assay (ELISA) was performed as previously described [25]. The purpose of this experiment is to assay the serum antibody specific for our *gidA* mutant STM strain. Serum IgG1 and IgG2a from mice inoculated with sterile PBS or the *gidA* mutant STM strain was measured 7 and 42 days post-immunization by ELISA as previously described [10]. High-binding flat-bottom ELISA plates (Thermo Fisher Scientific, Rochester, NY) were coated with 1 μg/ml of capture antibody (anti-IgG1 or anti-IgG2a) (Bethyl Laboratories Inc., Montgomery, TX) diluted in 0.05 M carbonate/bicarbonate buffer (pH 9.6) for 1 hour at room temperature. The wells of the microtiter plate were washed five times with washing buffer (50 mM Tris, 0.14 M NaCl, and 0.05% Tween 20) and blocked with blocking buffer (50 mM Tris, 0.14 M NaCl, and 1% bovine serum albumin [BSA]) overnight at 4°C. After washing, sera from both groups of mice were diluted in sample buffer (50 mM Tris, 0.14 M NaCl, 1% BSA, and 0.05% Tween 20) and the Mouse Reference Serum (Bethyl Laboratories Inc.) was diluted two-fold starting at a concentration of 1000 ng/ml and used for plotting the standard curve. After one hour incubation at room temperature, the plates were washed five times with washing buffer, and incubated for an additional hour at room temperature after the addition of a 1:250,000 dilution of horseradish peroxidase (HRP)-conjugated goat anti-mouse IgG (Bethyl Inc.) to the wells of the microtiter plate. After washing five times, 3, 3’, 5, 5’ tetramethylbenzidine (TMB) substrate was added to visualize antigen-antibody reactions. The reaction was stopped with 0.18 M H_2_SO_4_, and the optical density was measured at 450 nm.

### Lymphocyte proliferation assay

The lymphocyte proliferation assay was performed using the described method [26]. Splenocytes harvested on day 7 and 42 post-immunization were used in the lymphocyte proliferation assay. After harvesting, live splenocytes were determined by the trypan blue exclusion technique and counting with a hemocytometer. Cells from both groups of mice were plated in a 96-well U-bottom microtiter plate (Corning Inc., Corning, NY) at a cell density of 2 x 10^5^ cells/well. The cells were treated with STM cell lysate (1 μg/ml) and incubated at 37°C with 5% CO_2_ for 48 hours. The STM cell lysate was created from a WT STM 14028 culture that was grown to an optical density (O.D.)_600_ of 1.0, washed twice with PBS, lysed by sonication, and quantitated using a Bradford Assay. The percentage of cell survival was determined using the CytoTox-Glo Cytotoxicity Assay (Promega, Madison, WI). Quantification of viable cells was determined by the formula: Signal from Viable Cells = Total Cytotoxicity Signal – Initial Cytotoxicity Signal.

### Cytokine profiling

The cytokine profiling was performed using a commercially based multiplex assay as described [12]. Th1 (IL-2 and IFN-γ) and Th2 (IL-4 and IL-10) cytokine levels were determined from mouse sera at day 7 and 42 using a multiplex assay (Quansys Biosciences, Logan, UT). Cytokine production from splenocytes at day 7 and 42 was measured by plating splenocytes from both groups of mice in a microtiter plate at a cell density of 2 x 10^5^ cells/well. The cells were treated with STM cell lysate (1 μg/ml) and incubated at 37°C with 5% CO_2_ for 48 hours. The levels of Th1 and Th2 cytokines in the culture supernatant were determined using a multiplex assay (Quansys Biosciences).

### Passive transfer of cells and sera

Mice were bled for sera and splenocytes were harvested on day 42 post-immunization. Fifteen naïve mice were used with the mice being divided into three groups with five mice per group. Each group was inoculated via retro-orbital injection [27] with either 100 μl sterile PBS, 100 μl of sera from non-infected mice, or 100 μl of sera from mice immunized with the *gidA* mutant STM strain [28]. Another fifteen naïve mice were divided into three groups of five and each group was inoculated via retro-orbital injection [27] with 100 μl sterile PBS, 100 μl of splenocytes (1 x 10^7^ cells/100 μl) from non-infected mice, or 100 μl of splenocytes (1 x 10^7^ cells/100 μl) from mice immunized with the *gidA* mutant STM strain [28]. All groups were challenged by i.p. injection 24 hours later with a lethal dose (1 x 10^5^ CFU) of WT STM. Morbidity and mortality of these animals were monitored for 30 days after challenge. Mice suffering from lethal salmonellosis as determined by severe hunched posture, labored breathing, apathy, and ruffled fur were euthanized to prevent unnecessary suffering.

### Statistical analysis

Wherever appropriate, the data were analyzed using GraphPad Prism 5 software (GraphPad Software, San Diego, CA) and a Student’s *t* test. *P* values of ≤ 0.05 were considered significant, and data were expressed as arithmetic means with standard deviations. Animal mortality was analyzed using the Kaplan-Meier survival analysis with the log-rank (Mantel-Cox) significance test.

## Results

### Protective efficacy of the *gidA* mutant STM strain

**Figure 1:**
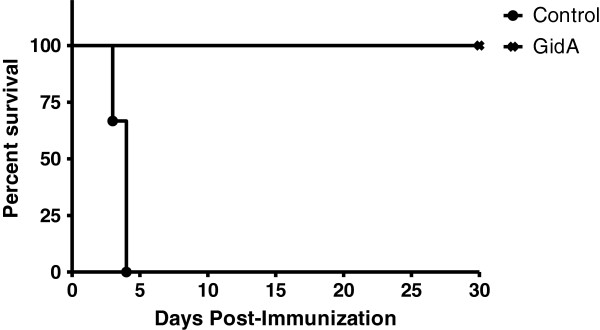
**Percent survival of mice immunized by i.p. injection with sterile PBS or 1 x 10**^**3**^**CFU of the *gidA *mutant vaccine strain, and subsequently challenged with a lethal dose (1 x 10**^**5**^**CFU) of WT STM on day 42 post-immunization.** Morbidity and mortality of these animals were monitored for 30 days after challenge. Full protection was provided to immunized mice while 100% mortality was seen in the control mice.

To examine the protection provided by GidA immunization, six BALB/c mice were i.p. injected with sterile PBS while another six mice were injected with 1 x 10^3^ CFU of the *gidA* mutant STM strain. AT 42 days post-immunization, all twelve mice were challenged with a lethal dose (1 x 10^5^ CFU) of WT STM. All of the control mice challenged with the WT STM strain died within four days of challenge. Meanwhile, all of the mice immunized with the *gidA* mutant STM strain survived the lethal dose challenge of WT STM. Furthermore, none of the mice immunized with the *gidA* mutant STM strain showed any lack of mobility, hunched posture, or ruffled fur associated with septic shock (Figure 1).

### Splenic bacterial counts after immunization

**Figure 2:**
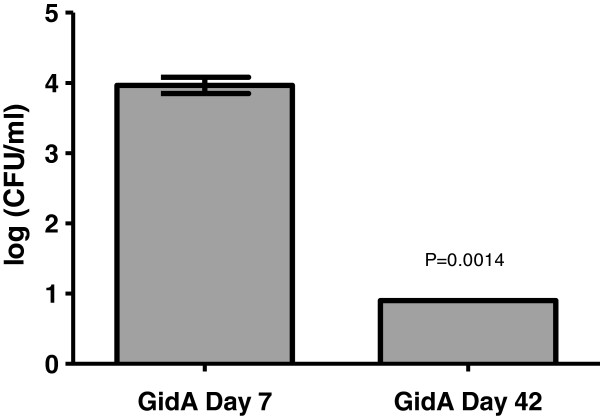
**Determination of bacterial counts in the spleen of mice immunized with 1 x 10**^**3**^**CFU of the *gidA *mutant vaccine strain.** Aliquots (0.1 g) of spleen were homogenized, serially diluted, and plated out on SS and LB agar to determine bacterial counts. The *P* value of 0.0014 shows a significant decrease in bacteria on day 42 post-immunization when compared to day 7 post-immunization. No bacteria were recovered from the spleens of the control mice.

We previously reported the level of bacteria recovered from spleens of mice inoculated with the *gidA* mutant STM strain was significantly less than that recovered from spleens of mice inoculated with the WT STM strain [12]. In this study, the *in vivo* stability of the *gidA* mutant STM strain was determined by examining its ability to colonize the spleen at Day 7 and at the time of challenge (Day 42). The number of viable bacteria recovered from mice immunized with the *gidA* mutant STM strain was 4.0 logs on day 7 post-immunization. At day 42 post-immunization, viable bacteria were still recovered from the spleen at 0.9 logs (Figure 2). The long persistence of the bacteria in mouse splenic tissues could enable sustained immune response activities in mice immunized with the *gidA* mutant STM strain. As shown by the histopathological grading in our initial GidA study, the colonization of the *gidA* mutant STM strain only caused mild necrosis with moderate infiltration of inflammatory cells in the spleen with little to no induction of proinflammatory cytokines and chemokines [12].

### T cell analysis in mice immunized with the *gidA* mutant STM strain

To determine whether T cells were activated in BALB/c mice immunized with 1 x 10^3^ CFU of the *gidA* mutant STM strain, isolated splenocytes from control and immunized mice were harvested at day 7 and 42 post-immunization. Splenocytes from both groups of mice were stained with antibodies against CD4 or CD8 in combination with anti-CD44 and anti-CD62L antibodies. These markers are used to distinguish naïve from activated or memory T cells [29]. The level of CD4^+^ cells were higher in the immunized mice (21.3%) when compared to the control mice (16.1%) on day 7 and again on day 42 (28.1 and 23.5%, respectively). There was no difference in the CD8^+^ populations between the control and immunized mice on day 7 and 42. Furthermore, on day 7 and 42 post-immunization, there was no significant difference between the control and *gidA* mutant immunized mice in the percentage of CD44^+^ and CD62L^+^ in both CD4^+^ and CD8^+^ T cells (data not shown).

### Serum IgG levels in mice after immunization

**Figure 3:**
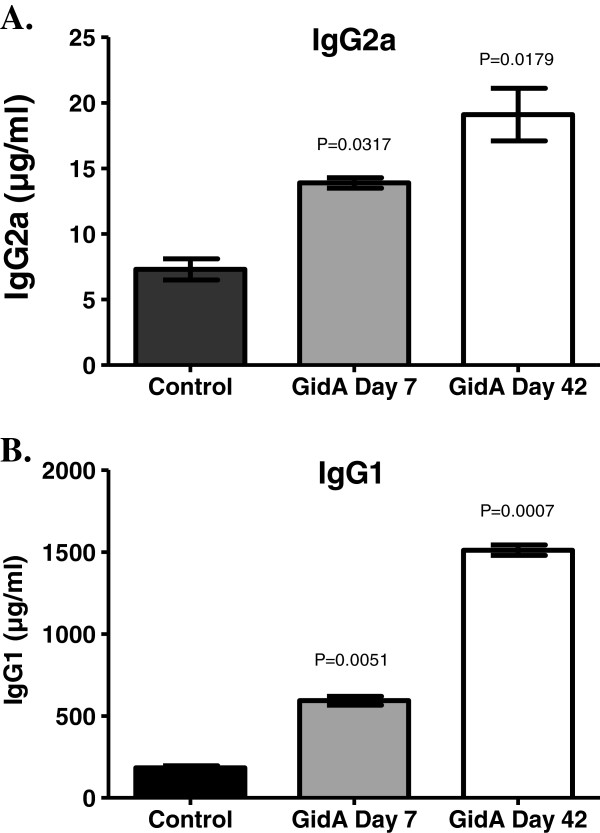
**BALB/c mice were immunized with 1 x 10**^**3**^**CFU of the *gidA *mutant vaccine strain or sterile PBS.** Serum IgG2a (**A**) and serum IgG1 (**B**) concentrations were determined by ELISA at the indicated times after immunization. The actual *P* values are provided comparing the sera levels of the immunized mice to that of the control group.

The *Salmonella* whole cell ELISA displayed a high-level of *Salmonella* specific antibody. In order to further characterize the immune response elicited after immunization with the *gidA* mutant STM strain, the sera of control and immunized mice were examined for the production of IgG2a and IgG1 antibodies as markers of Th1 and Th2 subsets, respectively. These findings indicate a significant increase in both IgG2a [*P*=0.0317 and *P=* 0.0179 for GidA day 7 and 42, respectively, compared to the control] and IgG1 [*P*=0.0051 and *P* =0.0007] in the sera of mice immunized with the *gidA* mutant STM strain with the highest levels being assayed on day 42 post-immunization. Furthermore, the IgG1 response, indicative of Th2, was higher in the immunized mice than the IgG2a response level in the immunized mice (Figure 3).

### Lymphocyte proliferation assay

**Figure 4:**
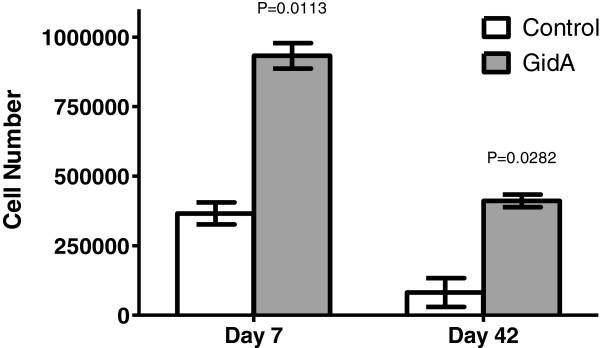
**Lymphocyte proliferation assay displaying the survival of splenocytes from control and immunized mice before and after treatment with STM cell lysate.** The actual *P* values for the given time points are provided showing the significant increase in proliferation in splenocytes from immunized mice in comparison to splenocytes from control mice.

Splenocytes harvested from control mice and mice immunized with the *gidA* mutant strain were used to examine the cellular immune response against treatment with STM cell lysate. At day 7 post-immunization, the splenocytes from the immunized mice displayed a significant proliferative response (932,590) [*P*=0.0113] compared to the splenocytes from the control mice (365,910). Once again, at 42 days post-immunization, the splenocytes from the immunized mice showed a significantly higher proliferative response (411,177) [*P*=0.0282] than the splenocytes from control mice (81,574) when treated with STM cell lysate. In contrast, splenocytes from non-immunized control mice showed little proliferation in response to treatment with the STM cell lysate (Figure 4).

### Cytokine analysis

**Figure 5:**
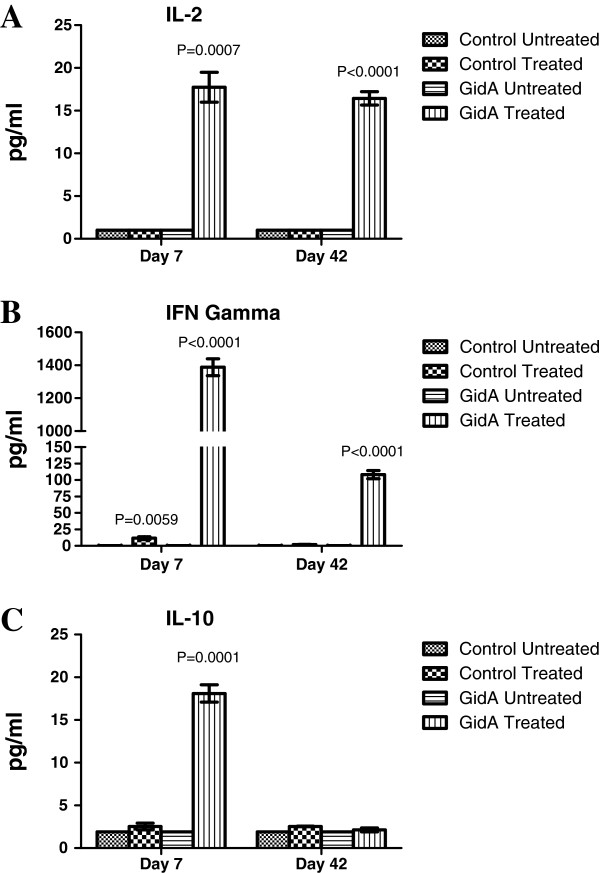
**Induction of IL-2, IFN-**γ**, and IL-10 in the cell culture supernatant from control and immunized mice before and after treatment with STM cell lysate.** Splenocytes were collected from both groups of mice at days 7 and 42 post-immunization and the levels of IL-2 (**A**), IFN-γ (**B**), and IL-10 (**C**) was determined using a multiplex assay. The actual *P* values are given for each time point.

Sera and splenocyte cell culture supernatants were examined for both Th1 (IL-2 and IFN-γ) and Th2 cytokines (IL-4 and IL-10). The sera of mice immunized with the *gidA* mutant STM strain showed no difference from that of the control sera in the level of cytokine induction on days 7 and 42 post-immunization (data not shown). These data confirm the findings in our initial GidA study which showed a marked reduction in the levels of all of the major cytokines when compared to sera of mice infected with the WT STM strain [12]. In the cell culture supernatant, the induction of Th1 and Th2 cytokines were significantly increased when GidA splenocytes were induced with STM cell lysate. Meanwhile, there was little to no cytokine induction in the cell culture supernatant when splenocytes from control mice were treated with the STM cell lysate. Furthermore, there was no IL-4 induction in either the control or GidA groups at days 7 and 42 (data not shown). On days 7 and 42 post-immunization, there was no difference between the treated and untreated control groups in the level of IL-2 induction. The level of IL-2 induction, however, significantly increased in the GidA treated cells (Figure 5A) *P*=0.0007 and *P* <0.0001]. The level of IFN-γ displayed a slight increase in the control treated cells (11.8 pg/ml) over the untreated control cells (0.3 pg/ml) on day 7, but showed no difference on day 42. In contrast, the GidA treated cells showed a marked increase in IFN-γ induction (1388.4 and 108.2 pg/ml) *P* <0.0001 and *P=*0.0001] compared to the untreated GidA cells (0.3 and 0.3 pg/ml) on days 7 and 42, respectively (Figure 5B). The levels of IL-10 were similar between the control groups on day 7, but the level of IL-10 induction in the GidA treated cells were significantly higher than that of the GidA untreated cells *P*=0.0001]. On day 42, there was no difference in IL-10 induction in either the control or GidA group (Figure 5C). Together, these results indicate that immunization with the *gidA* mutant STM strain elicited a mixed Th1 and Th2 response when treated with STM cell lysate.

### Protective efficacy of cells and sera

**Figure 6:**
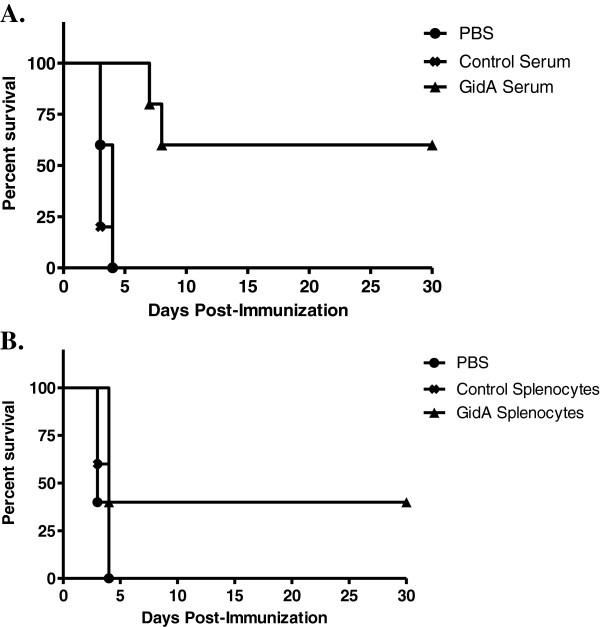
**Mice were immunized with 1 x 10**^**3**^**CFU of the *gidA *mutant vaccine strain or sterile PBS. Serum and cells were collected 42 days later and transferred to groups of five naïve mice.** All recipient mice were challenge by i.p. injection with 1 x 10^5^ CFU of WT STM 24 hours after transfer. Morbidity and mortality of these animals were monitored for 30 days after challenge. The serum passive transfer (**A**) was statistically significant with a *P* value of 0.0414 while the cell passive transfer (**B**) was not statistically significant. Statistical significance was calculated using the Kaplan-Meier survival analysis with the log-rank (Mantel-Cox) significance test.

A passive-immunization study was performed in order to evaluate the roles of antibody and cell mediated immunity provided by immunization of mice with the *gidA* mutant STM strain. Spleen lymphocytes (1 x 10^7^ cells/100 μl) or 100 μl of pooled sera taken from immunized mice or controls was administered by retro-orbital injection into groups of five naïve mice. Another group of five naïve mice was injected with sterile PBS to serve as an additional control. Approximately 24 hours later, all mice were challenged with a lethal dose (1 x 10^5^ CFU) of the WT STM strain. All of the mice receiving control sera, control cells, or sterile PBS died within four days of being challenged by the WT STM strain. The sera transferred from the *gidA* mutant immunized mice protected three of the five naïve mice from challenge. Furthermore, the two mice in this group that died showed a delay in death (7 and 8 days following challenge) when compared to the control serum and PBS control groups (Figure 6A). The cells transferred from the *gidA* mutant immunized mice protected two of the five naïve mice from challenge. The three mice that died from this group died in the same time period as mice receiving control cells and PBS (Figure 6B). From these data both parts of the immune response are somewhat protective, but antibody mediated immunity appears to be the more crucial of the two in protecting mice from WT STM.

## Discussion

In this study, for the first time, the mechanism of protection provided by immunization with the *gidA* mutant STM strain was characterized. GidA was originally thought to be involved in cell division due to the filamentous morphology observed when the cells were grown in rich medium supplemented with glucose [13]. More recent studies done in *E. coli* have shown GidA modulates several bacterial factors by a post-transcriptional mechanism to modify tRNA by the addition of a cmnm group at the 5 position of the wobble uridine (U34) of tRNAs [15-19,30,31]. It has been proposed that tRNA modification can serve as a regulatory mechanism to modulate gene expression[32]. Furthermore, it has been suggested that secreted proteins are particularly vulnerable to U34 hypomodification, and many codons in bacteria require proper U34 modification for efficient decoding [33]. Studies will need to be conducted in *Salmonella* to see if GidA modifies tRNA in the same fashion as in *E. coli.* Such studies are currently underway in this laboratory.

Immunization of mice with the *gidA* STM mutant strain provided full protection from a lethal dose challenge of WT STM. All of the immunized mice survived a lethal dose challenge, while all the naïve mice died within 4 days of challenge. Furthermore, none of the immunized mice displayed any visual signs of illness or septic shock associated with *Salmonella* infection. We chose to challenge the immunized mice with a WT STM dose of 1 x 10^5^ CFU which is highly lethal. In our initial GidA study, this dose was approximately 1000 times higher than the LD_50_ of the WT STM strain [12]. We chose such a high challenge dose because we feel it is more reflective of the amount of *Salmonella* animals are exposed to in the environment.

Antibody responses are known to contribute to *Salmonella* immunity [34-36]. It has been proposed that antibodies made by IgM memory B cells are the first-line defense mechanism against all infections and these antibodies are the only defense against T cell-independent antigens [37]. Studies in B cell deficient mice have shown that B cells are required for efficient protection from both primary and secondary *Salmonella* infection [36]. Our data indicates a strong humoral response to immunization with the *gidA* mutant STM strain. The Th2 marker, IgG1, showed a marked increase in sera of mice immunized with the *gidA* mutant STM strain. Naïve mice receiving sera from immunized mice were more protected than naïve mice receiving a passive transfer of cells from immunized mice. Further, the level of the Th2 cytokine IL-10 showed a significant increase in induction when splenocytes from immunized mice were treated with STM cell lysate. The strong Th2 response, however, was not accompanied by an increase in IL-4 induction. IL-4, along with IL-10, induces differentiation of uncommitted T cells toward a Th2 phenotype [38,39]. One possible explanation for this could be reasoned from the study by Okahashi et al. In their study, IL-4 knockout mice which were unable to generate classical Th2-type responses were still capable of producing significant antibody responses to inoculation with *Salmonella*[40].

Since *Salmonella* is a facultative intracellular pathogen, cellular immune responses are considered to be a crucial component of protective immunity. Protective cellular mediated immunity is mediated by CD4^+^ cells which results in the activation of macrophages and delayed-type hypersensitivity responses. Numerous gene target studies have shown the importance of CD4^+^ activation in resistance to *Salmonella* infection [41,42]. Our data indicates a cellular immune response in mice immunized with the *gidA* mutant STM strain. Although the flow cytometric analysis showed no induction of memory T cells, or difference in CD8^+^ cells, it shows an increase in CD4^+^ population in the immunized mice at both day 7 and 42 post-immunization. It has been shown that CD4^+^ cells are more important than CD8^+^ in resistance to *Salmonella* infection [43,44]. The passive transfer of cells to naïve mice from immunized mice did not confer full protection, and was not as significant as the serum passive transfer, but there was enough cell mediated immunity activated to protect a portion of the mice from a lethal dose challenge. Furthermore, splenocytes from immunized mice proliferated at a much higher rate than splenocytes from control mice when treated with STM cell lysate. The IgG1 induction was significantly more prominent than the induction of IgG2a, but the level of IgG2a was still significantly higher in the immunized mice than in that of the sera of the control mice. Furthermore, the induction of the Th1 cytokines, IL-2 and IFN-γ, shows a strong indication of cell mediated immunity induced by immunization. In particular, IFN-γ showed a marked increase in cell culture supernatant when splenocytes from immunized mice were treated with STM cell lysate.

The general consensus is that the ideal *Salmonella* vaccine should generate both humoral and cell mediated immunity. This is due to protective immunity to *Salmonella* in mice being attributed to a balance between humoral and cell mediated immunity with an emphasis on development of the Th1 and Th2 subsets [45,46]. In this study, the *gidA* mutant vaccine strain generated both Th1 and Th2 immunity with the Th2 immune response being the more prominent of the two. This was somewhat surprising since *Salmonella* is a facultative intracellular pathogen. One possible explanation for this could be found in our initial GidA study comparing the *gidA* mutant to the WT STM strain. The *gidA* mutant showed an approximate 1000-fold reduction in the ability to invade T84 intestinal epithelial cells, as well as a marked reduction in ability to cause systemic infection in mice. Additionally, transcriptional and proteomic profiling identified a significant down-regulation in numerous genes and proteins responsible for invasion. Overall, the *gidA* mutant vaccine strain provides full protection to mice when challenged with a highly lethal dose of WT STM. The passive transfer experiments show the importance of both humoral and cell mediated immunity in this protective mechanism. This is an initial study in which a proof of principle of protective immunity has been established suggesting a *gidA* mutant STM strain could be a good candidate for use in a live-attenuated *Salmonella* vaccine. Future studies will be conducted using oral immunization in order to establish the optimal immunization route. Once the immunization route is established, further studies will be conducted in a target host animal to determine efficacy and long-term protection. Based on our initial data, we believe a *gidA* mutant STM strain used in a live-attenuated vaccine could provide superior protection against highly lethal levels of *Salmonella* by stimulating humoral, cellular immunity and potentially mucosal immunity.

## Conclusions

Immunization with the *gidA* mutant STM strain provided full protection from a lethal dose challenge of WT STM. Sera levels of IgG2a and IgG1 were significantly higher in immunized mice when compared to sera of control mice, and the level of IgG1 showed a marked increase over IgG2a in the sera of immunized mice. Naïve mice receiving sera and cells from immunized mice were only partially protected from a lethal dose challenge of WT STM with the sera being more protective than the cells. A lymphocyte proliferation assay showed a marked response of splenocytes from immunized mice to treatment with STM cell lysate. Furthermore, the Th1 (IL-2 and IFN-γ) and Th2 (IL-10) cytokines showed a significant increase in the cell culture supernatant of splenocytes of immunized mice when treated with STM cell lysate. These data indicated the *gidA* mutant vaccine strain protects mice by inducing humoral and cellular immune responses with the humoral immune response being the primary mechanism of protection.

## Competing interests

The authors disclose no conflicts of interest.

## Authors’ contributions

DS participated in the design of the study, carried out the experimental work, performed the statistical analysis, and drafted the manuscript. AF designed and coordinated the study, and edited the manuscript. All authors read and approved the final manuscript.
